# Smog, Cognition and Real-World Decision-Making

**DOI:** 10.15171/ijhpm.2018.105

**Published:** 2018-11-04

**Authors:** Xi Chen

**Affiliations:** ^1^Department of Health Policy and Management, Yale School of Public Health, New Haven, CT, USA.; ^2^Yale Department of Economics, New Haven, CT, USA.

**Keywords:** Air Pollution, Cognitive Performance, Intelligence, Decision-Making, Cognitive Aging

## Abstract

Cognitive functioning is critical as in our daily life a host of real-world complex decisions in high-stakes markets have to be made. The decision-making process can be vulnerable to environmental stressors. Summarizing the growing economic and epidemiologic evidence linking air pollution, cognition performance and real-world decision-making, we first illustrate key physiological and psychological pathways between air pollution and cognition. We then document the main patterns of air pollution affecting cognitive test performance by type of cognitive tests, gender, window of exposure, age profile, and educational attainment. We further extend to a review of real-world decision-making that has been found to be affected by air pollution and the resulting cognitive impairments. Finally, rich implications on environmental health policies are drawn based on existing evaluations of social costs of air pollution.

Ninety-two percent of the world’s population lives in places where air quality levels exceed World Health Organization (WHO) limits set by WHO Ambient Air Quality Guidelines.^1^ Even in developed countries, more than 40% of Americans live in counties with unhealthy levels of air pollution,^2^ and 9 out of 10 European city-dwellers are exposed to pollution in excess of WHO guidelines.^3^ While it is well known that environmental stressors, such as polluted air, pose a significant threat to human health, knowledge about their consequences on our brains, especially among older adults, is limited. Damage on brains may affect earnings and health, impede human capital from being an important engine of economic growth, which in turn generate large economic losses we currently ignore.^4,5^

## 1. Air Pollution and Cognition: Key Pathways

Medical studies point to *physiological* pathways through which air pollution may impair cognitive ability,^6^ especially in the case of fine particulate matter (PM2.5). The small size of PM2.5 allows it to remain airborne longer, to carry toxins through small passageways, to penetrate buildings, to be inhaled easier, and to reach and accumulate within brain tissue.^7^ Post-mortem analysis detects that people living in more polluted areas for long periods tend to have elevated concentrations of PM2.5 in their brains, smaller brain volume, and higher rates of brain infarcts or areas of necrosis.^8^ Accumulation over time can be linked to markers of neuroinflammation and neuropathology, leading to symptoms of Alzheimer disease, one of the most prevalent and expensive forms of cognitive decline, and other forms of dementia.^9-13^ Even healthy people living in polluted environment with APOE Ɛ4 allele (known to increase risk of developing Alzheimer’s) demonstrate compromised cognitive responses compared with those carrying *APOE* gene with Ɛ3 allele. This gene environment interaction has been verified ranging from children to older adults.^14,15^ It is estimated that air pollution may account for 21% of dementia cases worldwide.^15^

Some other *physiological* pathways include: first, pollution exposure may increase risk for strokes and then vascular dementia^16^; second, people breathing polluted air are more likely to be oxygen deficient, which in turn impairs their cognitive abilities^17^; third, exposure to pollution leads to the growth of white-matter lesions, potentially inhibiting cognition^18^; fourth, air pollution may degrade cognitive ability via asthma and respiratory problems, persistently constraining the production of human capital, such as schooling^19^ and labor force participation^20^; finally, air pollution may also damage the immune system, hinder neurological development, and impair neuron behavior, which all contribute to long-term memory formation.^16^

Recent economic studies also show air pollution may disrupt cognitive functioning through *psychological* pathways. For example, high concentration of pollutants is significantly associated with headache,^21^ cause psychiatric distress,^22^ and increase the risk of feeling unhappy and depressed in the United States,^23^ Canada,^24^ and China.^25^ PM2.5 is currently the only pollutant among key atmospheric pollutants in the Air Quality Index (AQI) evidenced to cause psychological disorders.^26^

## 2. Air Pollution and Cognitive Test Performance: Key Patterns

Our central nervous system has 2 important tissues: gray matter and white matter. Gray matter represents information processing centers, and white matter represents the networking of – or connections between – these processing centers. Mathematics abilities, which require more local processing, mainly depend on gray matter. While language skills, which require integrating and assimilating information from distributed gray-matter regions in the brain, mainly rely on white matter.^27,28^

Air pollution may demonstrate differential effects on different types of cognitive tests. A large body of literature has proven that air pollution can reduce the density of white matter more than that of grey matter in the brain,^6,29^ which may explain why air pollution appears to affect both verbal and math skills but in the meantime has a larger effect on verbal test than on math test scores. A recent study in China overcomes several key challenges to identify the causal link between air pollution and cognitive ability, including math and language skills, across all ages beginning at 10 years old.^30^ They exploit individual level longitudinal data, precisely matched timing and geographic location of the tests, rich demographic controls, exogenous changes in air pollution exposure, and information on sorting behavior. They indeed find that exposure to air pollution lowers both verbal and math test scores of survey subjects, and the former effect is larger than the latter.

The effect of air pollution on cognition also has a gender component. Brain scanning studies reveal that men have larger amount of gray matter* activated* during general intelligence tests than women do, but women have more white matter *activated* during general intelligence tests than men do.^31^ Given that gray matter is more required by math tests and white matter is more required by verbal tests, it is predicted that men’s cognitive performance, especially in the verbal domain, tends to be more affected by exposure to air pollution, while women’s cognition performance, especially in the math domain, is likely to be more affected. A more recent study explores the impact of air pollution on older adults in China.^32^ They indeed find that the detrimental effect on verbal skills is more concentrated towards older males.

Moreover, the effect of air pollution on cognition is cumulative and may become larger as one ages. An investigation using a national sample of older adults in China finds that the detrimental effect is the largest for the older persons above age 65. Even within the 2 older cohorts, the effect on those above age 65 is twice as much as those in the age cohort 55-64.^32^ A 12-unit worsening of 3-year average AQI is linked to an equivalent of 1 year huge loss in educational attainment for those who are above age 65.^32^ Since people in old age often make critical financial decisions and spend most of their wealth in their lifetime, the more sizable effect to the elderly and the associated large economic loss are especially worrisome.

The finding of larger negative effect of cumulative than transitory exposure to air pollution provides policy implications that short-term interventions (eg, wearing face masks or turning on air filters on polluted days, rescheduling high-stakes cognitive activities) may be less effective than improving long-term air quality. Public policies should effectively clean up the sky, rather than merely investing in avoidance. The finding of substantial cumulative effect also suggests that improvement in cognitive ability may take long time to realize, especially for many who have been exposed to many years of heavy pollution.

The fact that people who receive less education or often work outdoors demonstrating larger effects alerts us that environmental stressors may enlarge social inequality, including cognitive ability.^30^ Future research needs to evaluate to what extent this impact can be intergenerational. Once the social costs on environmental justice are accounted for, policymakers will have better understanding about the real cost of air pollution to help tighten existing environmental standards.

## 3. Air Pollution, Cognition, Suboptimal Decision-Making and Associated Costs

Three channels may provide a preferential foundation linking haze, cognitive impairment to real world decision-making in a set of important contexts, such as job market, health insurance market, stock market, and even the decision to commit a crime.

First, cognitive impairment may increase the cost of cognitive processing required to make decisions. Dementia and other forms of cognitive decline increase a set of financial mistakes regarding credit behavior as people age.^33^ Medicare beneficiaries diagnosed with dementia are less likely to comprehend key institutional features of insurance markets and more likely to make suboptimal decisions.^34^

Assembling 15 years of administrative Medicare records on 7.4 million people age 65 and above, tracking their health, onset of Alzheimer disease and related forms of dementia, demographics, residential cumulative exposures to fine particulate air pollution (PM2.5), and financial decisions, a recent study finds evidence that higher cumulative exposure increases the rate of dementia.^35^ Specifically, an 1 μg/m^3^ increase in *decadal* exposure to PM2.5 (8.5% of the mean) increases the probability of a dementia diagnosis by the end of the decade by 0.5 to 1.2 percentage points (4% to 6%). Even more modest change in air pollution measured by a 1 μg/m^3^ rise in annual average concentrations of PM2.5 would increase the rate of dementia by 1% to 3% (around 100 000 to 300 000 cases), lowering direct medical expenditures on dementia by $3.5 to $10.5 billion per year in 2017 dollars. There is, however, no evidence that exposure to PM2.5 affects the diagnosis rates for morbidities thought to be unrelated to air pollution, providing some convincing evidence against confounding.

Meanwhile, higher cumulative exposure raises the probability to make poor financial decisions in prescription drug insurance markets under Medicare Part D among those not diagnosed with dementia.^36^ In particular, a 1 μg/m^3^ increase in average *decadal* exposure to PM2.5 increases potential savings by $4 (a 1% increase relative to the mean) and the probability of choosing a drug plan that is dominated by another in terms of cost, risk protection, and quality by 0.25 to 0.43 percentage points (a 0.5% to 1.2% increase relative to the mean). These effects are 3% to 6% of the size of the negative effect of a dementia diagnosis on the same decision-making outcomes. Even more moderate change in air pollution measured by a 1 μg/m^3^ rise in annual average concentrations of PM2.5 would cost all consumers more than $60 million per year additional spending on prescription drugs under Medicare Part D.

In addition to complex decision-making like health insurance choices, hourly and daily spikes in pollution are found to reduce white-collar workers’ labor productivity in cognitive tasks, such as number of calls that call center workers in a travel agency complete each day.^5^ The decrease in cognitive productivity is driven by increases in time spent on breaks rather than the duration of phone calls. Among various pollutants, PM2.5 poses the greatest threat to contemporaneous cognition, in part because it penetrates buildings and pollutes indoor air.

Second, air pollution may undermine rational risk attitudes and patience when discounting in a remote time horizon.^37^ Bad air quality in Manhattan therefore substantially reduces same-day return to the S&P 500.^38^ Individuals may also become more aggressive, more selfish, and less cooperative with greater sense of fairness.^39^ Violent crime rate in Chicago is higher when air pollution is heavier, which reflects the impulsive nature of violent crime.^40^

Third, since cognitive impairment can reduce life expectancy, it may reduce investments in health capital, leading to additional chronic conditions that increase the complexity of decisions about health insurance.^41^ More generally, the potential return to the decisions can be health-state dependent and therefore decline in worsened cognitive status.^42^

## 4. Policy Implications

Most of the existing evidence on air pollution, cognitive impairment and decision-making comes from the United States and other developed countries where air pollution concentration is very low. The implications for developing countries with more polluted air can be quite large and deserve more attention in future research. The profound economic and health implications of air pollution on poor cognitive functioning suggest that the costs of pollution can be largely underestimated. The comprehensive set of evidence we summarize underscores the need for stronger regulations around air quality in response to the increasing concerns on air pollution, in particular the hidden cost on intellect and decision-making, which has not been well recognized before.

Such large social costs can be avoided if individuals demonstrate strong willingness to pay for improved air quality. An average Chinese resident is willing to pay $88 per year (or 3.8% of annual income) for a 1 μg/m^3^ reduction in PM2.5, while an average US resident is willing to contribute as high as $891 (or 2.1% of annual income).^43^ Although people in the United States on average seem to be willing to pay a much higher amount in absolute terms, Chinese residents are more willing to pay a larger share of their income.^23,43^ When we obtain most reliable values of willingness to pay for major population groups in each country, policy makers will be able to learn the potential demand and how they can be matched with the supply of good air for cognitive activities and beyond.

The establishment of the US Environmental Protection Agency (EPA) and the Clean Air Act of 1970 (1970 CAA) authorized the development of comprehensive federal and state regulations to substantially reduce air pollution, such as through creating and enforcing the national Ambient Air Quality Standards (AAQS). However, the AAQS has only been based on narrowly defined health assessments. Realizing the severity of this issue, the EPA recently calls for more research to assess the impact of ambient air pollutants on central nervous system function, such as cognitive processes and addresses the adequacy of existing standards. Many countries in the world have been adopting the AAQS as their environmental standards. This recent move in EPA’s strategy to address air pollution may therefore generate spillover effects on more stringent environmental standards and effective regulations in other countries.

As one of the most polluted countries adopting the AAQS, China has substantially tightened its environmental regulations in the past five years. In 2013, China started to implement the national *Air Ten Plan*, which involved ten concrete measures to restrict air pollutant emissions. Since then, air pollution in Chinese cities has dropped by an average of 30%.^44^ While such achievement may bear important implications for other countries which aim to improve their environmental quality, and neighboring countries may directly benefit from lower level of pollution being transmitted to them, the large uncertainty of governmental policies may put the these efforts and their effectiveness at high risks.

The prospect on such advancement in environmental quality is grim. In August 2018, The US President Donald Trump announced plans to dramatically relax fuel efficiency standards, a move that would flood American communities with more dangerous airborne particulates from vehicles.^45^ Moreover, the EPA determined that Trump’s new regulations for emissions at coal-burning power plants will cause an additional 1400 deaths a year.^46^

Small-scale schemes are useless if other countries fail to act. Dirty air in many countries is swept over from neighboring countries. As a key global health issue, no wall could be built to effectively shield people from external pollutants. Countries should work closely together to take global actions in their fight against air pollution, one of the largest avoidable causes of death, illness, and human capital degradation.

## Acknowledgements

This study was supported by James Tobin Fund at Yale Economics Department, Yale Macmillan Center Faculty Research Award, the PEPPER Center Scholar Award (P30AG021342), and other NIH/NIA grants (K01AG053408; R03AG048920).

## Ethical issues

Not applicable.

## Competing interests

Author declares that he has no competing interests.

## Author’s contribution

XC is the single author of the paper.
